# Augmenting Mental Health in Primary Care: A 1-Year Study of Deploying Smartphone Apps in a Multi-site Primary Care/Behavioral Health Integration Program

**DOI:** 10.3389/fpsyt.2019.00094

**Published:** 2019-02-28

**Authors:** Liza Hoffman, Emily Benedetto, Hsiang Huang, Ellie Grossman, Dorosella Kaluma, Ziva Mann, John Torous

**Affiliations:** ^1^Department of Primary Care, Cambridge Health Alliance, Cambridge, MA, United States; ^2^Division of Digital Psychiatry, Department of Psychiatry, Beth Israel Deaconess Medical Center, Boston, MA, United States; ^3^Department of Psychiatry, Cambridge Health Alliance, Cambridge, MA, United States

**Keywords:** mental health, mobile apps, smartphone apps, integrated primary care, behavioral health integration, mental health integration

## Abstract

**Background:** Integrating behavioral health (BH) services into primary care is an evidence-based intervention that can increase access to care, improve patient outcomes, and decrease costs. Digital technology, including smartphone apps, has the potential to augment and extend the reach of these integrated behavioral health services through self-management support impacting lifestyle behaviors. To date, the feasibility and acceptability of using mental health mobile apps within an integrated primary care setting has not yet been explored as part of routine clinical care.

**Objectives:** The objectives of this study were to (a) test the feasibility of using mental health applications to augment integrated primary care services; (b) solicit feedback from patients and providers to guide implementation, and (c) develop a mental health apps toolkit for system-wide dissemination.

**Methods:** Cambridge Health Alliance (CHA) is a safety-net healthcare system that includes three community hospitals and 12 Primary Care (PC) clinics serving nearly 150,000 ethnically and socioeconomically diverse patients around Boston. To select and disseminate mental health apps, a four-phase implementation was undertaken: (1) Evaluation of mental health mobile applications (2) Development of an apps toolkit with stakeholder input, (3) Conducting initial pilot at six primary care locations, and (4) Rolling out the app toolkit across 12 primary care sites and conducting 1-year follow-up survey.

**Results:** Among BH providers, 24 (75%) responded to the follow-up survey and 19 (83%) indicated they use apps as part of their clinical care. Anxiety was the most common condition for which app use was recommended by providers, and 10 (42%) expressed interest in further developing their knowledge of mental health apps. Among patients, 35 (65%) of participants provided feedback; 23 (66%) reported the tools to be helpful, especially for managing stress and anxiety.

**Conclusions:** Our findings indicate mental health apps are applicable and relevant to patients within integrated primary care settings in safety-net health systems. Behavioral health providers perceive the clinical value of using these tools as part of patient care, but require training to increase their comfort-level and confidence applying these tools with patients. To increase provider and patient engagement, mobile apps must be accessible, simple, intuitive and directly relevant to patients' treatment needs.

## Introduction

Integrating behavioral health care services into primary care is an evidence-based intervention that can increase access to care, improve patient outcomes, and decrease costs (1). The need for integrated behavioral health (BH) is driven by the heavy burden of mental health conditions, including depression, which is now recognized by the World Health Organization as the leading global cause of disability (2), as well as insufficient numbers of specialized behavioral health clinicians like social workers, psychiatrists, psychologists, and case managers. Key elements of integrated care models include screening for high prevalence mental health conditions, integration of behavioral health clinicians into primary care settings, collaborative team building, and care management for patients (3). With ~40% of mental health care in the United States delivered in the primary care setting, the demand for integrated BH services has outpaced availability (4).

Like other areas of healthcare, digital technology has been proposed as a means to augment and extend the reach of BH integrated care services. These technologies can be classified into three categories: (1) patient facing, (2) primary care facing, and (3) virtual visits (5). Patient facing technologies to extend integrated care may include smartphone apps that offer self-management or guided interventions like Cognitive Behavioral Therapy. Primary care facing technologies include tools to support increased capacity and confidence to offer mental health treatment such as consultation platforms, tele-mentoring, and clinical decision support for integrated care teams. Virtual visits reflect the ability to offer telemedicine services to support integrated care. While there is a growing evidence base and clinical experience with using both virtual visits and primary care facing technologies (5) there is limited to no research or clinical evidence for using smartphone apps to support integrated care.

The lack of knowledge on incorporating mental health-related smartphone apps into integrated care systems is especially surprising considering the potential of mobile technology in mental health. Numerous survey studies have demonstrated those with mental health conditions own smartphones and are interested in using apps for their care (6–8). Meta-analysis of randomized clinical studies of smartphone apps for depressive symptoms (9) and anxiety disorders (10) have demonstrated early efficacy data. In many cases, consumers with mental health conditions are not waiting for the evidence to catch up; today over 10,000 mental health related apps are available from the iTunes and Android app stores (11). Case reports and clinical experience support that people are using these apps to learn about their mental health, track symptoms, and self-manage their condition (12, 13). However, exploring the utility of these apps outside of research studies remains largely uncharted.

Prior efforts transitioning promising mental health research apps into actual clinical care settings underscores the need for careful assessment. One substance use disorder app that showed promising evidence to reduce frequency of alcohol consumption (14) was later found to be difficult to support and maintain in actual clinical settings without the additional support and structure offered in the research setting (15). Reviews of smartphone app adoption by healthcare professionals, beyond mental health and integrated care settings, suggest that perceived barriers can include concerns about privacy, legal ramifications, cost, workload, and need for increased information technology (IT) support (16). Despite barriers, there is evidence that apps are being introduced to care. For instance, research from the United Kingdom suggests that already 30% of mental health providers recommend apps to patients (17). On the patient side, there is also concern that many commercially available mental health apps may not be designed for those with more severe symptoms (18).

In this study, we sought to provide early clinical data from both the patient and clinician perspective on the usage and integration of mental health apps in a busy integrated BH care service at Cambridge Health Alliance (CHA), a safety net healthcare system that includes three community hospitals and 12 Primary Care (PC) clinics which serve nearly 150,000 ethnically and socioeconomically diverse patients in the greater Boston area (19). The healthcare system had implemented an integrated model of care, based on the collaborative care model (20) and Screening, Brief Intervention and Referral to Treatment (SBIRT) (21), to support screening, assessment, and treatment of behavioral health conditions in primary care clinics. The integrated BH staff provide evidence-based brief clinical interventions focusing on patients primarily experiencing low to moderate anxiety, depression, and substance use disorders. Consult psychiatrists provide diagnostic clarification and management strategies for the primary care team. Integrated psychotherapists provide brief treatment (6–8 sessions) grounded in evidence-based treatment modalities including Cognitive Behavioral Therapy (CBT), Problem-Solving Therapy (PST), Behavioral Activation (BA), and Mindfulness Based Stress Reduction (MBSR). Care managers work primarily with patients experiencing low to moderate anxiety and depression; they use interventions including Motivational Interviewing (MI) and BA to support patients in reaching health and lifestyle-related goals and to bridge patients to BH providers. To answer the question of whether mental health apps can be adopted within routine BH care and serve as a helpful tool for patients, we present the results of one institution's experience evaluating, piloting, and disseminating mental health apps across an integrated primary care system.

## Methods

### Step 1: Selecting Apps

The first step in introducing apps into care was to identify a series of existing apps that could be recommended to patients receiving integrated care services. Given that the vast majority of these apps fall outside of the Food and Drug Administration (FDA) regulation and there is no formal evaluation or gold standard for judging these apps, we assembled a seven-person multi-disciplinary team of integrated therapists, care managers, administrative and Informational Technology (IT) leadership, and patients to identify and evaluate mental health self-management apps over the course of 10 months. We sought apps that offered patient facing interventions, avoided direct use with a clinician, were free, and claimed to provide privacy protections for patient generated data—understanding there is no set of gold standard apps or criteria to pick apps. Thus, we focused on apps that were: (1) aligned with clinical interventions utilized in our program; (2) developed by a “trusted” source such as medical, academic, and/or research institutions, and/or companies with clinical consultants; (3) free to download, and (4) contained privacy statements at the time of our evaluation. Drawing from our workgroup's clinical knowledge and experience caring for CHA's safety-net patient population, we also considered apps' accessibility in terms of our patients' language needs, literacy levels, and alignment with treatment goals (22). In seeking such apps, we read select literature to help orient us to broader findings and challenges in the mHealth space (23), consulted with app researchers and designers, and sought feedback from our clinical teams and the patients they serve. Without validated app evaluation tools to guide our selection process, we relied on expert consensus throughout the four-stage study process to select a set of apps that best supported our clinical interventions and the needs of our patient population.

### Step 2: Creating a Toolkit Around Apps

We identified an initial list of 13 applications and sought guidance from our institution's Patient Partner Lead who represents CHA's patient partners, a group of patient volunteers who serve on quality improvement teams to advise on key projects. Drawing on feedback from our Patient Partner Lead, staff member expertise, papers on app evaluation (24), and elements of the user version of the Mobile Apps Rating System (U-MARS) (25), the workgroup next narrowed the list to nine apps and drafted a process for introducing the apps through the Primary Care Behavioral Health Integration program (PCBHI) in Primary Care. In drafting the process for introducing these apps to patients, we aimed to incorporate several elements: (a) suggestions to staff as to when to introduce the apps; (b) appropriate language for staff to use in discussing the apps with patients, and (c) brief descriptions of the apps in patient-facing materials. The evaluation team and Patient Partner Lead co-designed a set of tools for introducing the BH apps, including a patient-facing summary sheet describing the risks/benefits of using smartphone applications and a mobile apps “toolkit” that contained descriptions of the purpose and main features of the nine selected apps. We then trained six care managers to identify patients who might benefit from using mobile apps and introduce the tools to patients (including clinical rationale, product demonstration, goal setting, and plan for follow-up). Care managers were given additional training around discussing app privacy/security with patients and reviewed all points detailed within the patient-facing summary sheet. As a quality improvement project to improve access to high quality self-management tools within primary care, this implementation study was not subject to formal approval by our Institutional Review Board as per Cambridge Health Alliance's ethics guidelines and national regulations. Before introducing the selected mobile apps to patients, care managers obtained informed verbal and written consent from all pilot participants and no personally identifiable information was collected during the course of the pilot.

### Step 3: Initial Pilot Testing

Before disseminating the apps toolkit across our integrated care system, the team conducted pilot-testing over 2 months at the six primary care clinics where the trained care managers were located. During the pilot, patients were referred to care managers by consult psychiatrists, integrated therapists, and primary care providers through “warm handoffs” or the real-time transfer of patients from one provider to another. When demonstrating mobile applications, care managers used iPads available in the clinic or their own smartphones (with appropriate security measures in place). After ~2–4 weeks, care managers followed up with patients either in person or by phone to conduct structured interviews in which patients were asked the following questions: Did you use the app? Did you find it to be helpful? Do you have any additional comments?

Following the formal conclusion of the pilot, care managers continued to gather feedback related to patients' experience over the course of 1 year. Preliminary themes were identified through review of all available data, several group discussions leading to consensus in our multidisciplinary team, and updated with data gathered in the larger roll out as described below.

**Figure 1:**
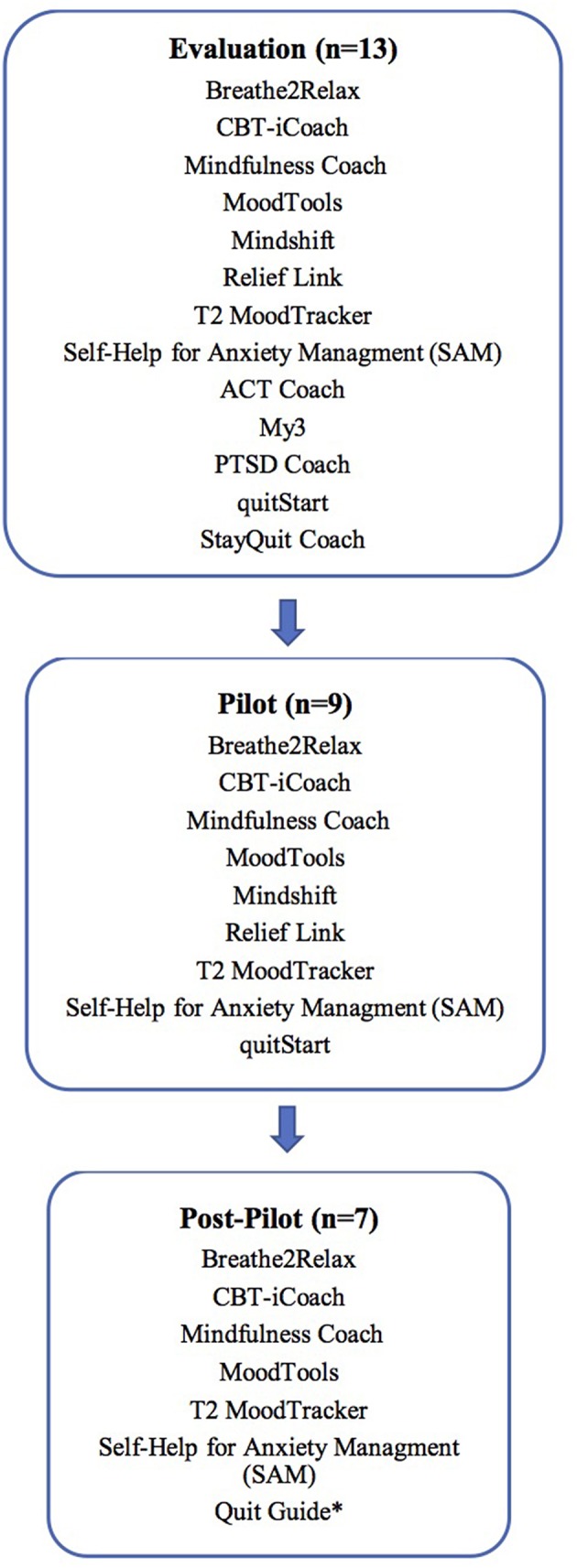
App selection across stages of the study. *Quit guide replaced quitStart with post-pilot input from CHA's tobacco treatment team.

Following the pilot test, we evaluated patient and care manager feedback and adapted procedures to roll out the program to all 12 of our primary-care practices. Our final toolkit contained seven apps as we removed two apps from the list of nine used during the pilot. As smartphone apps are dynamic and always changing, during this phase of the study new information about a smoking app became available that led our team to substitute for one from the pilot—reflecting the real-world challenge of using apps in health care (see Figure 1).

### Step 4: Staff Training and App Dissemination

To facilitate use of apps by staff, we wrote two electronic medical record (EMR) standardized “smart phrases” which could be incorporated into patient-facing after-visit summary documents. These “smart phrases” contained the list of recommended self-management apps and the patient-facing summary sheet explaining risks/benefits of using mobile apps. Through a series of staff meetings, we trained all integrated BH staff (therapists, consult psychiatrists, care managers) on best practices for utilizing mental health apps within clinical care. Because of the pilot testing and early staff engagement in the project, many were already familiar with the basic premise of the project and supportive of its goals.

One year after staff training and dissemination of CHA's mobile apps toolkit across all 12 primary care clinics, we followed up by sending a survey to integrated BH staff. In this survey, we asked questions exploring (a) staff utilization of apps; (b) perceived clinical impact, and (c) experience introducing apps to patients.

## Results

### Quantitative Patient Experience

Over the 2-month duration of the pilot intervention, care managers introduced mental health apps to 56 adult patients. Fifty-four (96.4%) patients agreed to use the tools while 2 (3.5%) patients declined (1 due to security concerns and 1 due to time constraints). Patients in the pilot were 18–70 years old with a mean age of 36.5 years, and 37 (66.1%) were female. English was not the language of medical care for 6 (10.7%) patients; 4 (7.1%) primarily spoke Portuguese and 2 (3.5%) primarily spoke Spanish. The tools were introduced to 35 (62.5%) patients for anxiety and stress-related issues and to 20 (35.7%) patients for depression. Eight (14.2%) patients received apps to target sleep issues and 3 (5.3%) patients were introduced to apps for other issues. Thirteen (23.2%) of the 56 patients were introduced to the tools for multiple conditions/issues.

**Figure 2:**
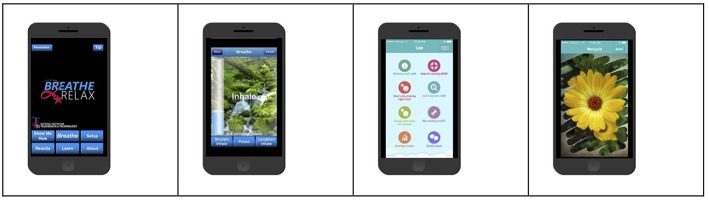
Screenshots of Breathe2Relax (Left two) and Self-Help for Anxiety Management (Right two).

The care managers were unable to follow-up with 19 patients during the 2-month pilot period. Of the 35 (64.8%) patients who provided feedback on usage of the tools, 7 (20%) patients were unable to use the tools (3 due to equipment limitations, 4 for other reasons). Twenty-three (65.7%) of patients who provided feedback found the apps to be helpful. Twenty-four (68.5%) of patients reported using Breathe2Relax and 24 (68.5%) reported using Self-Help for Anxiety Management (SAM). Patients used Breathe2Relax to help them sleep, to manage anxiety, and to prepare for stressful or mentally taxing situations. When using SAM, patients tended to access just a few of the features that focused on symptom relief, education about anxiety, strategies for managing strong emotions, and mood tracking. (Figure 2)

### Qualitative Patient Experience

After the 2-month pilot and over the course of the following year, care managers collected general feedback from patients that is presented in Table 1 as a combination of paraphrasing and direct quotes. Review of this feedback by our multidisciplinary team and discussion yielded consensus around 7 broad themes: ease of use, educational value, role in self-monitoring and enhancing self-awareness, relaxation/mindfulness, ability to incorporate into daily life, data and security concerns, and value of staff role in encouraging engagement with mental health apps.

**Table 1:** Key themes and illustrative quotes from the 1 year study.

**Theme**	**Illustrative quote**
Ease of use	“I like that this app is simple and easy to navigate”
	“I do the breathing exercise every time I get into the car. I also shared it with my boyfriend who speaks Portuguese. We do the exercise together.”
	“I use this app when I'm waiting in the car at my daughter's school”
	“I like short exercises. I can use them in different places”
	“I can do a walking meditation during my lunch break”
Education	“The TED talks were helpful”
	“I re-read the education sections as a reminder”
	“I like being told I'm not crazy”
	“It was helpful learning more about anxiety and coping thoughts”
Self-monitoring and Self-awareness	“I want to track my mood so I can show the data to my therapist. I can't always remember how I felt during the week”
	“I like data. Recording my mood every day helps me to know I'm doing ok”
	“I track my mood before and after I do an activity. The next time I think about exercising, I can look back to see how I felt afterwards”
	“The sleep diary helps me to keep track of my sleep patterns. It's easier than trying to remember later on”
	“I record how I'm feeling and also what's making me anxious”
	“I had a hard time identifying my thoughts. Maybe I could focus on this in therapy?”
	“Doing the exercise helped me to recognize how tense and anxious I can be”
	“When I use the app, it's easier for me to size up my thoughts. I can then figure out whether they're trivial or beyond my control”
Relaxation/Mindfulness	“The progressive muscle relaxation exercise helps me to fall asleep”
	“I enjoyed the exercise focusing on one sensation. I think this app could be helpful to many patients”
	“The app was calming and relaxing”
	“I love the app and guided relaxation for mindfulness”
	“I use the guided imagery exercise with a student at my school. We do it every day during nap time. It helps him and it helps me too!”
	“It used to take me hours to fall asleep. Now it takes me 7 min”
Difficulty incorporating into daily life	“I don't always remember to use the app when I'm stressed”
	“Sometimes I forget to use it. I tell myself I'm going to do it later and then I forget”
	“I looked at the app, but I didn't use it on a regular basis”
	“Apps are overwhelming when I'm stressed. I need to get away from technology”
Data and security concerns	“I worry about my virtual image. I'd feel more comfortable using an app from CHA that is protected in the same way my EMR is protected”
	“I don't have space on my phone”
	“I'm worried about my data”
Staff support	“It was helpful learning about the different parts of the app. I downloaded it in the past, but I didn't understand the point of the exercises”
	“Having you check in with me helped to keep me on track”

### Quantitative Staff Experience

After 1 year of the intervention (which involved 32 integrated BH staff), 24 (75%) completed the follow-up survey. Within the integrated BH team roles, nine (100%) care managers, 12 (75%) psychotherapists, and 3 (43%) consult psychiatrists responded. The number of responses to each survey item varied.

Twenty-three (95.8%) BH staff members answered the survey question regarding their utilization of mobile tools with patients; nineteen people (82.6%) indicated that they incorporate BH apps into their clinical work. Five (25%) BH staff members out of 20 indicated that they introduce apps to patients 25–50% of the time, and 9 (45%) introduce mobile tools <25% of the time. When using apps with patients, the condition addressed most often by BH staff was anxiety 20 (83.3%), followed by stress 18 (75%), depression 14 (58.3%), alcohol use 6 (25%), and tobacco use 6 (25%). Of the 23 (95.8%) staff members who reported on their experience using the CHA's mobile app toolkit, the apps most commonly used were Breathe2Relax, Mindfulness Coach, and Self-Help for Anxiety Management (see Table 2 below). The survey also assessed techniques and strategies staff employed in introducing apps to patients: 19 (79.1%) reported discussing access/interest/ability, 14 (58.3%) discussed clinical basis for recommending that app and how it connects with the patient's goals, 14 (58.3%) demonstrated how to use the app, 13 (54.1%) helped the patient download the app, 13 (54.1%) followed up and discussed app use with patients at later visits, 12 (50%) set a follow up plan for app use, 9 (37.5%) discussed security and privacy risk associated with app use, and 7 (29.1%) created an action plan around app use.

**Table 2:** Apps most frequently recommended by BH staff.

**Survey Item: Which apps from CHA's mobile app toolkit do you introduce to patients?**	**Reponses (*N* = 23) *n* (%)**
Breathe2Relax	14 (61)
Mindfulness coach	12 (52)
SAM	10 (43)
CBT-iCoach	9 (39)
Moodtools	5 (22)
T2Moodtracker	3 (13)
QuitforLife	3 (13)

BH staff found a variety of benefits and challenges to incorporating apps into clinical care (see Table 3) and 10 out of 24 BH staff (42%) expressed a need for more practice and training on using each tool within the CHA's mobile app toolkit.

**Table 3:** Staff views on the benefits and challenges of utilizing BH apps.

**Survey Item**	**Response (*N* = 23) *n* (%)**
**IN GENERAL, WHAT DO YOU THINK ARE THE BENEFITS OF INCORPORATING APPS INTO CLINICAL CARE? (SELECT ALL THAT APPLY)**	
Patients have the opportunity to practice self-management skills between meetings	15 (65)
Using apps enhances care provided by therapist and primary care provider	13 (57)
Patients' ability to manage mood/anxiety/substance use improves when using apps	13 (57)
Patients are excited to try self-management tools	11 (48)
Patients feel more confident in managing their BH conditions independently	9 (38)
**IN GENERAL, WHAT DO YOU THINK ARE SOME CHALLENGES TO INCORPORATING APPS INTO CLINICAL CARE? (SELECT ALL THAT APPLY)**	
My patients do not have enough data on phones to use apps	6 (26)
I do not have enough time during my sessions to introduce self-help tools	5 (22)
I am unfamiliar with apps' content and functionality	5 (22)
I do not feel comfortable navigating apps and demonstrating use to patient	5 (22)
My patients are not interested in using apps	2 (8)
I do not believe they would be helpful for my patients	0 (0)
My patients are concerned about data security	0 (0)
I am unsure how to assess whether a patient could benefit from using an app	0 (0)

### Qualitative Staff Experience

Consult psychiatrists, therapists, and care managers were also asked to provide qualitative/free-text feedback on apps in the 1 year follow-up survey. Representative responses for the three most utilized apps are presented below in Table 4 alongside patient feedback. Feedback concerning clinical use, accessibility, patient engagement/motivation, and other areas is presented below in Table 5.

**Table 4:** Top three apps utilized by staff during the 1 year study (as reported through the staff survey).

**App**	**Developer**	**Description**	**Popular features**	**Patient feedback**	**Staff feedback**
Breathe2Relax	Defense health agency connected health (formerly the National Center for Telehealth and Technology)	Developed for military personnel. Contains step-by-step instructions for diaphragmatic breathing, provides a visually engaging audio guided breathing exercise, and enables users to record their stress levels using a tracking function. http://t2health.dcoe.mil/apps/breathe2relax	Guided diaphragmatic breathing exercise	“I have been doing it every day. I feel like it is cleaning my brain. I have been suggesting it to my friends. I learned that problems we will always have, but I feel more patient now. I noticed some changes on my self-esteem I am becoming more optimistic. I used to have mind racing and not anymore”	“I like Breathe2Relax's visuals” “Breathe2Relax is more popular because it is easier - no language barrier” “Breathe2Relax is very useful as it contains education about deep breathing, a basic exercise and the option to personalize settings”
Self-Help for Anxiety Management (SAM)	The University of the West of England	Developed for college students with low to moderate anxiety. SAM is a “platform of interactive, therapeutic, and well-being exercises” meant to assist users with learning about anxiety, cognitive reframing, relaxation, distress tolerance, lifestyle change and self-monitoring of physical and emotional states related to anxiety. http://sam-app.org.uk	“Picture Peace” “Stop That Thought” “It's Only a Thought” Anxiety Tracker Anxiety Education	Picture Peace: “I slow down and think about what's bothering me” Stop That Thought: “I noticed my thoughts going from “this is annoying” to “I've made a horrible mistake taking this job”. Writing my thoughts down makes me realize how absurd they are and banishing them in the app helps me to better reframe the situation and get more perspective”	“For SAM, I use the picture peace, stop that thought, and it's only a thought features most often” “Patients really like the picture peace feature; I use it too!”
Mindfulness coach	VA's National Center for PTSD and DoD's National Center for Telehealth & Technology	This app was developed to help Veterans, Service members, and others learn how to practice mindfulness. It offers exercises, information, and a tracking log so that you can optimize your practice. https://mobile.va.gov/app/mindfulness-coach	Guided mindfulness exercises	“I used to get jammed up about small things. Doing these exercises helped me to realize that burnt pizza isn't the end of the world”	“Mindfulness coach is great, but I wish it were also available for android. I like the guided mindfulness better than on moodtools especially if it is for a patient not with depression”

**Table 5:** Organizing themes and illustrative quotes from staff survey.

**Theme**	**Illustrative quote**
Clinical use	“For CBT-iCoach, I use the “quiet your mind” tools that contains progressive muscle relaxation exercises and guided imagery”
	“Before the app, I tried to do sleep diaries with patients on paper. They were mostly unsuccessful since the patient would lose the paper or forget to complete it. With the app, patients have access to their phones all the time and can send reminders to themselves”
	“Great way to have patients practice exercises between sessions; both provider and patient happy to have concrete tool”
	“Apps are containing when patients feeling stressed; some are fun to use”
	“The positive is that the app is free and easy to utilize, the downside is that a patient may spend too much time engaging on their phone and not building relationships with others/community. The young adult patients enjoy the apps, while the older patients want to belong to a community, i.e., yoga, tai chi, or meditation class”
	“I don't use them very often because I do a lot of guided relaxation in session and have my patients record them on their phone. Usually I think this kind of approach is best because it allows me to make the exercise specific to their needs. That some of the apps are helpful for specific skills like breathing with the metronome (if that is what it is called I am not sure)”
	“Sometimes I think my training in behavioral medicine allows me to create a different tool with the patient that is more specific to them”
	“I like PTSD Coach and Insight Timer as well”
	“The simple practice of using the app(phone) as a way to relax and take a mindful minute”
	“I use them very often. Mostly Breathe2Relax and SAM”
Accessibility	“To me, it is very difficult to incorporate the apps because they are only in English”
	“Some apps too complicated to explain quickly; patient seems overwhelmed”
	“Sometimes it takes too long to download an app with the patient, sometimes they need to come and see me for a second time to practice together”
	“Definitely language is a barrier for some of them”
Patient engagement & motivation	“Patients are generally receptive to the idea. Most that have put it into practice have been practicing mindful breathing, some are still having trouble maintaining ongoing use in order to derive any benefit”
	“Most of them don't end up downloading it/using it”
	“Patient only used app one time to learn deep breathing exercise, but continued practicing deep breathing before bed without assistance of app”
	“Usually I do not have negative feedback but I notice a good number of patients mentions they did not continue using in home. Sometimes they say: “I forgot to use it.” Maybe because this area is still new for patients? I am not sure”
	“Patients like it- they like that they have an option to do something on their own to look at after the visit”
	“Recently I had a good feedback from a patient (serious case of DV) that the breathing exercises was helping her a lot and she was even teaching her friends. I was impressed how excited she was about the exercises. Patient states feeling more calm, has been sleeping much better. She is still not completely free of nightmares but improved a lot. She used to have nightmares every day but this week had two times a week”
	“Usually I get good feedback from patients. I call them about 2 weeks after my visit and check with them but I do not have the info if they continue using for a long time”
Additional feedback	“Technology is in”
	“Re: question 7: training the PCPs about the tools; a lot of them don't know about it. They could introduce patients to tools then do a warm handoff to the care partner”
	“Instead of formal training, I think more case examples of how the apps were incorporated can be a helpful reminder about using the apps. I admit that since I see both kids and young adults, I sometimes forget about the many options available to someone 18+”

## Discussion

Our results of piloting the use of smartphone apps in a primary care setting with integrated BH staff demonstrate the feasibility, opportunity, and challenges of introducing mobile technologies into formal care settings. Through our program of mobile app evaluation, pilot-testing, and 1 year long local dissemination into routine practice we demonstrated that mental health apps can be introduced to patients of varying ages, literacy levels, and English-language proficiency. On the patient side, our results suggest that patients found the mobile tools helpful in learning about their mental health conditions, practicing relaxation skills, and monitoring mood symptoms. Barriers to use included technical limitations (e.g., lack of smartphone, limited data), difficulty incorporating into daily life, low motivation, and limited mental health app options for non-English speaking patients. On the clinical side, members of the integrated care teams were receptive to apps but used them with patients <50% of the time—with chief barriers including lack of time to discuss apps and being unfamiliar or uncomfortable with apps.

While our study results reflect the experience at a single healthcare system in an urban environment, several lessons learned in our experience are generalizable. Reflecting on both the successes as well as areas for further improvement in our development and rollout of apps for integrated care teams offers insights for others seeking to build their own app libraries, develop app toolkits, onboard staff, and engage patients.

### Selecting Apps and the Need to Update

Selecting appropriate apps to recommend for use in integrated care is a core element in a successful rollout, but also a moving target as these apps are constantly updating and evolving. Relying on websites or services that claim to curate apps and score them based on certain metrics is not helpful, as such scores do not take into account the unique needs of intended users (e.g., lower literacy levels), and do not reflect the simple reality that apps frequently update and change. We found that gathering a multidisciplinary panel to search, evaluate, and test apps was useful and practical. Such a panel should remain an ongoing and integral part of any app rollout and ideally meet at least quarterly to continue to update and evaluate new apps and new evidence. In terms of searching for new apps to support integrated care, we noted that apps selected in our search focused on helping patients learn more about their mental health conditions, enhance self-awareness, develop skills related to distress tolerance, and improve anxiety management. While we realize that today's mental health apps offer functionalities that can allow for real time monitoring and facilitate communication with the treatment team, such functionalities raise a number of complex questions related to risk/privacy/liability that could not be addressed within the scope of this quality improvement project.

### Building a Toolkit and the Need for More Hands-On Training or a New Digital Navigator

Although the app toolkit that we developed to help the clinical teams introduce, integrate, and support app use was critical to success, it was not sufficient in and of itself. Offering the clinical team a list of apps, core information and talking points on each app, and electronic medical record smart phrases to document app usage was key to the feasibility of the intervention. Clinical staff recommended apps that had a clear purpose, simple user-interface, and were easy to navigate and demonstrate within a brief period of time. When introducing Breathe2Relax, for example, staff were often able to review the technique of diaphragmatic breathing, help patients to personalize settings, and practice the app's guided breathing exercise in under 10 min. Mental health apps that have a clear purpose and that are easy to review during staff trainings may enhance BH staff confidence and efficiency in introducing these tools to patients and facilitate higher adoption across the primary care system.

However, we found that the clinical teams sought additional support in understanding how each app worked, in demonstrating apps to patients, and having enough time in clinical visits to discuss an app. This suggests two possible responses: either (a) further hands-on training for clinical staff or (b) the introduction of a new member of the care team in the form of a digital navigator. While there is little evidence to support optimal teaching strategies for app use, we propose that hands on training involving role playing introducing and using apps with patients may offer an effective means to help the clinical team feel more confident in using apps in care settings. Another option would be to refer patients to a new member of the care team called a digital navigator—much as we did in the initial pilot study with the care manager—who is an expert at setting up, teaching, and supporting use of apps. This is akin to referring a patient to a pharmacist on the team to learn more about medication or dietician to craft a customized healthy eating plan.

### Supporting Patients With Apps and Ensuring Apps Are Accessible

Patients in our study found apps useful and preferred those with clear purpose that had an engaging interface, were intuitive to use, and easy to navigate. The preference for simplicity aligns with findings within the field of user experience and design (26) as well as emerging research (27, 28) suggesting that apps containing a limited number of concepts and activities may be more appealing than those with a number of features. Apps containing limited text and simple/concrete language demand less from a cognitive standpoint (29) and may prove most accessible if patients are turning to apps during times of emotional distress when their cognitive abilities are compromised. Of note, the most popular feature within our mobile apps “toolkit” was “Picture Peace” a grounding exercise within Self-Help for Anxiety Management (SAM) that involves swiping across a blank screen to reveal a nature photo beneath. Patients described this feature as being “fun” and chose to use it over other exercises that were less visually pleasing and that demanded more from a cognitive standpoint.

However, the lack of apps available in multiple languages was also a limiting factor that made it impossible to use apps with some patients who may have benefited from this intervention. Several patients were also unable to run apps on their phone because of issues installing the apps or lacking sufficient memory on their smartphone to run the apps.

### Connecting to Care Through Apps

Our results also suggest new clinical applications for mental health apps beyond those studied and implemented in our study. Within our integrated primary care model, care managers often serve as a “bridge” to longer-term BH care, providing education about BH conditions, teaching basic distress-tolerance skills, and focusing on brief interventions related to lifestyle behavior change. In using mental health apps, patients can begin to develop greater insight into their thoughts, feelings, and behaviors, an essential component of any therapeutic treatment intervention. Providing patients with self-management apps while they are waiting for in-person BH services may also help patients to stay connected to their care team and cultivate a sense of “emotional holding” during this bridging period (29). These apps may also spread awareness and interest in BH services, as evidenced by multiple patients who shared a recommended app with friends and family because they thought it may be useful to them as well.

These same apps may help introduce patients to BH treatment who would have otherwise not been interested or declined to engage because of stigma, discomfort or misunderstanding about the field and treatments offered. Thus, the potential of population health enabled through apps and digital technology will remain a focus of our continued research. While our study did not explore potential cost-savings associated with our intervention, we plan to investigate the financial implications of digitally augmented integrated BH care in future studies.

### Limitations

Like all studies, ours has several limitations. As a real-world implementation study that sought to understand feasibility of app selection, uptake, and introduction into integrated care clinics—we were unable to control for many variables including patient severity, disease state, and clinician participation among others. In considering our patients' feedback that was not incorporated into a formal qualitative framework, we recognize that having care managers solicit feedback directly from patients may have influenced patient self-report and the subsequent interpretation of this data. Also given how often apps update and change—we realize that our results are difficult for others to replicate especially as we tailored our research to the local needs of the patients we serve. While we believe our study included a representative sample, it is difficult to quantify the number of types of patients, or clinicians, who are willing to engage and use apps as part of care. Further, we realize there are currently no gold standard apps, gold standard methods to select apps, or gold standard tools to quantify the impact of apps on care. Thus, we hope that the process and results of this present research can help guide others in taking the first steps to study the best ways to introduce digital technologies like apps into their clinical settings.

## Conclusions

Although there are barriers to implementing digital technologies like smartphone apps into integrated BH settings, with careful planning and stakeholder engagement they can be useful tools in real-world clinical settings. While apps alone are not a panacea to addressing limited access to mental health care and lifestyle interventions, when deployed thoughtfully, they can help extend care to support patients with mild and moderate behavioral health conditions on a personal level and communities on a population level.

## Author Contributions

All authors listed have made a substantial, direct and intellectual contribution to the work, and approved it for publication.

### Conflict of Interest Statement

The authors declare that the research was conducted in the absence of any commercial or financial relationships that could be construed as a potential conflict of interest.
